# Magnetic resonance cholangiopancreatography: Comparison of two- and three-dimensional sequences for the assessment of pancreatic cystic lesions

**DOI:** 10.3892/ol.2015.2935

**Published:** 2015-02-05

**Authors:** KEFU LIU, PING XIE, WEIJUN PENG, ZHENGRONG ZHOU

**Affiliations:** 1 Department of Radiology, The Affiliated Suzhou Hospital of Nanjing Medical University, Suzhou, Jiangsu 215008, P.R. China; 2 Department of Radiology, Fudan University Shanghai Cancer Center, Fudan University, Shanghai 200032, P.R. China; 3 Department of Oncology, Shanghai Medical College, Fudan University, Shanghai 200032, P.R. China

**Keywords:** magnetic resonance cholangiopancreatography, pancreatic duct, pancreatic cystic lesion, image quality, two dimensional, three dimensional

## Abstract

The present study aimed to compare two-dimensional (2D) and three-dimensional (3D) magnetic resonance cholangiopancreatography (MRCP) for the assessment of pancreatic cystic lesions. Between February 2009 and December 2011, 35 patients that had been diagnosed with pancreatic cystic lesions, which was confirmed by surgery and pathology, underwent pre-operative 2D or 3D MRCP for pre-operative evaluation. In the present study, the quality of these 2D and 3D MRCP images, the visualization of the features of the cystic lesions, visualization of the pancreatic main duct and prediction of ductal communication with the cystic lesions were evaluated and compared using statistical software. The 3D MRCP images were determined to be of higher quality compared with the 2D MRCP images. The features of the cystic lesions were visualized better on 3D MRCP compared with 2D MRCP. The same capability for the visualization of the segment of the pancreatic main duct was exhibited by 3D and 2D MRCP. There was no significant difference between the area under the receiver operating characteristic curve values of 2D and 3D MRCP, which assessed the prediction of communication between cystic lesions and the pancreatic main duct. It was concluded that, compared with 2D MRCP, 3D MRCP provides an improved assessment of pancreatic cystic lesions, but does not exhibit an improved capability for the visualization of the pancreatic main duct or for the prediction of communication between cystic lesions and the pancreatic main duct.

## Introduction

Magnetic resonance cholangiopancreatography (MRCP) has been widely used for the evaluation of the pancreatobiliary system. At present, numerous techniques, including three-dimensional (3D) and two-dimensional (2D) sequences, have been used in MRCP. Certain studies have compared 2D and 3D MRCP for the visualization of the pancreatobiliary system (1–12). However, studies that have compared the use of 2D and 3D MRCP for the visualization of pancreatic cystic lesions focused solely on intraductal papillary mucinous neoplasm (IPMN) (5,8). The two previous studies concluded that image quality of the pancreatic duct of 3D MRCP was superior to that of 2D MRCP, 3D MRCP identified the morphological details of IPMN with more confidence compared with 2D MRCP. In addition, there was no increase in the level of accuracy in predicting ductal communication of the lesion in 3D MRCP, and 3D and 2D MRCP performed similarly for predicting benign and malignant lesions. By contrast, certain case reports have revealed that serous and mucinous cystadenoma may communicate with the main pancreatic duct (13–16), which may impact the choice of treatment strategy. Pancreatic tumour enucleation has the benefit of minimal tissue trauma to the pancreas when compared with resectioning the tumour, adn therefore tumour enucleation is recommended for pancreatic cystic tumours <2–3 cm with non-adherence to pancreatic main-ducts (17). Therefore, pre-operative imaging evaluation of pancreatic cystic lesions is important.

In the present study, 2D and 3D MRCP were compared in terms of the ability to visualize pancreatic cystic lesions, including additional subtypes to IPMN.

## Materials and methods

### Patients

Between February 2009 and December 2011, 35 patients, consisting of 12 males and 23 females with an age range of 24–71 years (mean age, 48.7 years), underwent MRCP as a pre-operative evaluation. The patients all received a final diagnosis of pancreatic cystic lesions that was confirmed by surgery and pathology. The institutional review board of The Affiliated Suzhou Hospital of Nanjing Medical University (Suzhou, China) approved the present retrospective study and waived the requirement for informed consent. The final diagnoses were mucinous cystadenoma in eight patients, serous cystadenoma in nine, IPMN in eight patients, comprising four branch-type and four combined-type IPMNs, pancreatic retention cyst in three patients, pancreatic pseudocyst in four patients, simple cyst in two patients and cystic pancreatic splenosis in one patient.

Eight out of 35 patients were examined using only 2D MRCP, the remaining 27 patients were assessed using 2D and 3D MRCP. In total, 28 out of 35 patients had been evaluated using surgery or pathology to determine whether the cystic lesions communicated with the pancreatic main duct.

### MR technique

All MR imaging (MRI) examinations were performed on a 3.0-T MRI system with a torso phased array multicoil (Signa HDx; GE Healthcare Life Sciences, Chalfont, UK). There was a fasting period of at least four hours prior to imaging, and no oral contrast material or antiperistaltic agents were administered.

An axial T2-weighted single-shot-fast-spin-echo (SSFSE) sequence was used to localize the biliary and pancreatic ductal system. The parameters consisted of a repetition time of 1055 msec, echo time of 600 msec, bandwidth of 62.50 Hz, slice thickness of 6 mm and spacing of 1 mm, with the number of slices being 21. The MRCP protocol was composed of 2D radial coronal thick-slab breath-hold SSFSE and 3D respiratory triggering fast-recovery-fast-spin-echo (FRFSE) using the array spatial sensitivity encoding technique (ASSET).

The parameters for 2D MRCP were as follows. The repetition time was 10,000 ms, the echo time was 900 ms, the bandwidth was 62.50 Hz and the slice thickness was 50 mm, without spacing and with a matrix size of 352×352, field of view (FOV) of 30 cm, ASSET of 2.00 phase acceleration (PH). The number of radical slices was 10 and the partial radial spacing was 10°. The radial direction was clockwise, the breath-hold duration was five short breath-holds and there was fat saturation. The scan time of one slab was 1s. The breath-holds were performed at the end of inspiration, subsequent to two preceding full respirations. The thick-slab sequences were acquired using a radial loop centered at the level of biliopancreatic confluence. The first image of the radial loop was obtained from the posterior border of the right hepatic lobe.

The parameters for 3D MRCP were as follows. The echo time was min, the bandwidth was 62.50 Hz, the slice thickness was 1.8 mm and the number of images was 60, with no spacing. The zero filling interpolation was 2, the matrix size was 320×256, the FOV was 32 cm and the ASSET was 2.00 PH. The respiratory interval was 1, the trigger point was 30 and the trigger window was 30. There was an inter-sequence delay of 199 msec, a respiratory rate of 18–20 and there was fat saturation. The data were gathered at the end-expiratory phase.

### Imaging processing and evaluation

All source images were transferred to a workstation (Advantage Windows 4.3 CT Workstation; GE Healthcare Life Sciences). Post-processing of the source images obtained with the respiratory-triggered 3D FRFSE sequence was performed using multiplanar volume reformation with the maximum intensity projection. The angle and range of the section thickness were freely changeable by the assessor to visualize the pancreatobiliary system.

Two assessors that were blinded to the clinical history of the patients and the results of the evaluation provided the other observer, independently reviewed each image on a display monitor in a random order and evaluated the image quality using a five-point scale. Image quality was ranked as: 1, poor (non-interpretable); 2, suboptimal; 3, acceptable (minimal artifacts); 4, good; and 5, excellent (no artifacts).

The delineation of the head, body and tail of the pancreatic duct and the pancreatic cystic lesion were also evaluated using the following grading system: 5, excellent (complete delineation); 4, good (delineation of ≥90%); 3, fair (delineation of <90%); 2, poor delineation; and 1, not visualized.

The diagnostic confidence in whether there was communication between the cystic lesion and the pancreatic main duct was assigned on a five-point scale: 1, definitely absent; 2, possibly absent; 3, indeterminate; 4, probably present; and 5, definitely present.

### Statistical analysis

Statistical analyses were performed using SPSS 15.0 software (SPSS, Inc. Chicago, IL, USA). P<0.05 was considered to indicate a statistically significant difference. The comparison of the image quality between 2D and 3D MRCP were performed using the Mann-Whitney U test. The diagnostic capability of the 2D and 3D MRCP images for predicting ductal communication of the lesion was calculated by measuring the area under the receiver operating characteristic (ROC) curve (Az). Calculation of the statistical significance of the difference between the Az values for the 2D and 3D MRCP was performed using the Z-test. Analysis of the inter-observer agreement between the two readers was performed using the κ statistic.

## Results

**Figure 1:**
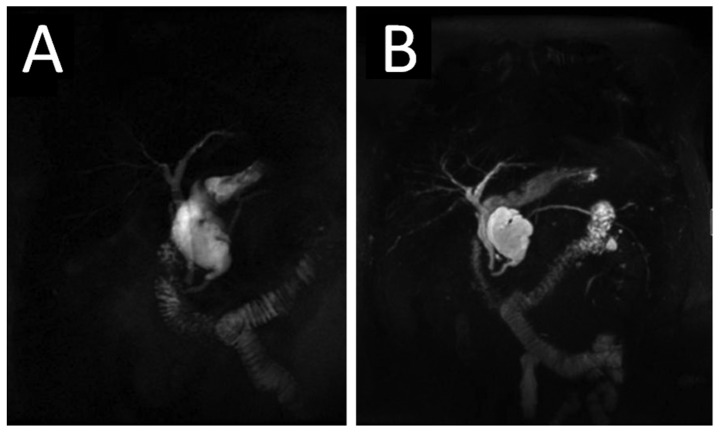
MRCP performed on a 61-year-old male diagnosed with pancreatic mucinous cystadenoma. (A) 2D MRCP clearly revealed that the cystic lesion communicated with the pancreatic main duct. (B) 3D MRCP exhibited superior image quality and features of the lesion compared with 2D MRCP, and also clearly revealed that the cystic lesion communicated with the pancreatic main duct. 2D, two-dimensional; 3D, three dimensional; MRCP, magnetic resonance cholangiopancreatography.

**Figure 2:**
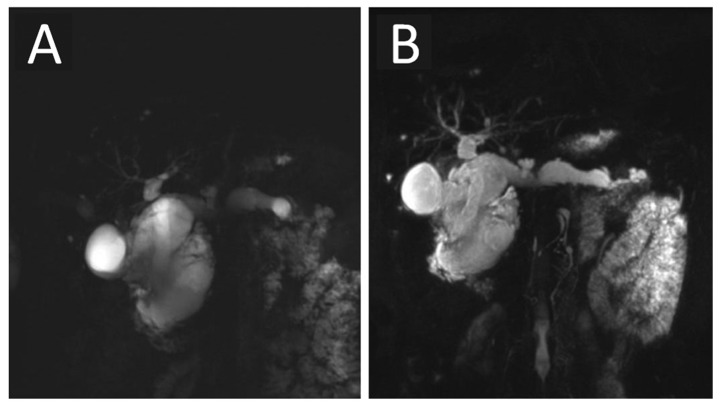
MRCP performed on a 71-year-old male diagnosed with pancreatic combined-type intraductal papillary mucinous neoplsam. (A) 2D MRCP clearly revealed that the cystic lesion communicated with the pancreatic main duct. (B) 3D MRCP exhibited the superior image quality and features of the lesion compared with 2D MRCP, and also clearly revealed that the cystic lesion communicated with the pancreatic main duct. 2D, two-dimensional; 3D, three dimensional; MRCP, magnetic resonance cholangiopancreatography.

For each reader, the image quality of 3D MRCP was judged to be higher compared with 2D MRCP (Table I; Figs. 1 and 2).

**Figure 3:**
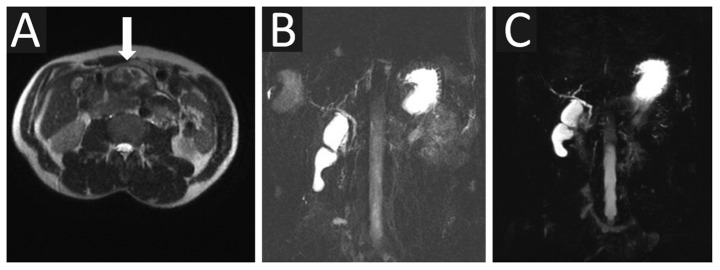
Imaging examination performed on a 35-year-old male diagnosed with pancreatic pseudocyst. (A) T2-weighted imags revealed that the pancreatic pseudocyst exhibited low signal intensity (arrow). (B) 2D MRCP did not reveal the cystic lesion. (C) 3D MRCP exhibited the superior image quality compared with 2D MRCP, but did not reveal the cystic lesion and pancreatic main duct. 2D, two-dimensional; 3D, three dimensional; MRCP, magnetic resonance cholangiopancreatography.

**Figure 4:**
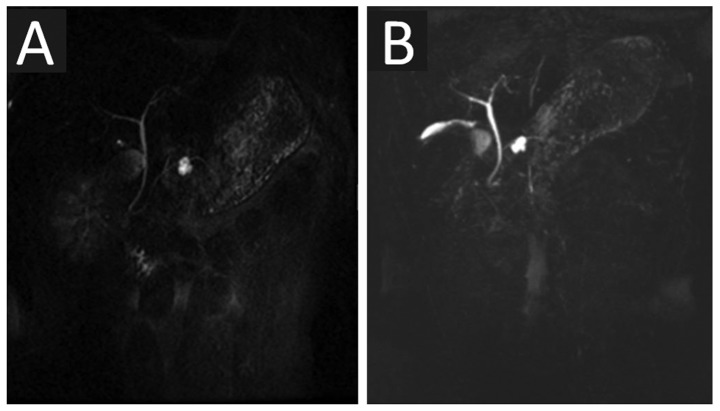
MRCP performed on a 46-year-old male diagnosed with pancreatic serous cystadenoma. (A) 2D MRCP resulted in the misdiagnosis of the cystic lesion communication with pancreatic main duct as the lesion was immediately adjacent to the main duct. (B) 3D MRCP exhibited the superior image quality, features of the lesion and the same capability of visualization of the pancreatic main duct in comparison with 2D MRCP, but ductal communication of the cystic lesion was misdiagnosed on 3D MCRP also. 2D, two-dimensional; 3D, three dimensional; MRCP, magnetic resonance cholangiopancreatography.

The cystic lesion features were visualized better on 3D MRCP compared with 2D MRCP (Table I; Figs. 1 and 2), but the cystic lesions were not visualized on MRCP in two out of eight cases that were examined using 2D MRCP only. These two lesions comprised one retention cyst and one pseudocyst. The cystic lesions were not visualized by MRCP in two out of 27 cases that were examined using 2D and 3D MRCP, comprising one splenosis and one pseudocyst. These lesions exhibited the same imaging features on T2-weighted imaging, with low or mixed-low signal intensity (Fig. 3). The same capability of visualizing the segment of pancreatic main duct was exhibited by 3D and 2D MRCP (Table II; Fig. 4).

The 2D and 3D MRCP Az values for the prediction of ductal communication between the cystic lesion and the pancreatic main duct involved 25 cases for 2D MRCP, due to the exclusion of seven cases that were not evaluated for communication and three cases that did not demonstrate cystic lesions on MRCP. The Az value for 3D MRCP involved 19 cases, due to the exclusion of seven cases in which the communication was not evaluated and one case in which MRCP did not reveal the cystic lesion. There was no statistically significant difference between the prediction of ductal communication with cystic lesions on 2D and 3D MRCP (Table III and Figs. 1 and 2). There were three cases in which MRCP led to the erroneous prediction of ductal communication with lesions, as these lesions were adjacent to the pancreatic main duct (Fig. 4).

## Discussion

In comparison with 2D thick-slab MRCP, 3D MRCP using ASSET exhibits superior image quality and the features of the cystic lesions are better visualized. The 3D sequence provides the merits of a high signal to noise ratio and intrinsically contiguous sections that may be used to reconstruct images in any projection, which yields the anatomical overview normally provided by thick-slab 2D MRCP images (4,5,8). The long acquisition time is the main disadvantage of primary 3D MRCP compared with the 2D MRCP technique. ASSET, which is applied to phase-encoded directions, overcomes this disadvantage of 3D MRCP, as ASSET allows for a higher matrix to be maintained without prolonging the imaging time (2). The FRFSE sequence can increase the signal to noise ratio by refocusing residual transverse magnetization into a final spin echo and using a −90° fast-recovery pulse to flip back along the z-axis to increase longitudinal magnetization and create a driven equilibrium (4).

In the present study, MRCP did not reveal the cystic lesions in two out of eight cases that were examined using only 2D MRCP. These lesions comprised one pancreatic retention cyst and one pseudocyst. By contrast, MRCP did not reveal the cystic lesions in two out of 27 cases that were examined using 2D and 3D MRCP, comprising one cystic pancreatic splenosis and one pseudocyst. These lesions exhibited the same imaging features in that the T2-weighted imaging demonstrated low or mixed-low signal intensity. As MRCP typically appears heavy on T2-weighted imaging, the lesion may be not revealed by MRCP if the lesion exhibits low signal intensity on the T2-weighted image.

In the present study, 3D MRCP exhibited the same capability for the visualization of the segment of pancreatic main duct as 2D MRCP, which is in agreement with the results of the study by Kim *et al* (12), Palmucci *et al* (3) and Sodickson *et al* (4), but other studies have reported varying results (2,5,18). The reasons for this may include that the pancreatic duct is sensitive to respiration (17) and that 2D MRCP exhibits superior in-plane resolution (4).

Previous studies have revealed that the ability of 2D and 3D MRCP to predict ductal communication with the cystic lesion does not significantly differ between the two MRCP techniques, although 3D MRCP exhibits a higher area under the ROC curve (5,8). In the present study, the 2D and 3D MRCP Az values for the prediction of communication between cystic lesions and the pancreatic main duct were not statistically different, either. Therefore, 3D MRCP requires additional technical improvement for the visualization of extremely small anatomical features.

The present study possessed certain limitations. First, the present study demonstrated a patient selection bias in that the majority of the studies performed MRCP upon the identification of indeterminate CT findings. The sample size was relatively small, and additional studies with an increased number of cases may be required in the future. In addition, as 2D and 3D MRCP were not applied together in all cases, the present study is not a paired analysis.

To conclude, in comparison to 2D MRCP, 3D MRCP provides an improved assessment of pancreatic cystic lesions. However, 3D MRCP does not exhibit an improved capability for the visualization of the pancreatic main duct and prediction of communication between cystic lesions and the pancreatic main duct.

## Figures and Tables

**Table I:** Comparison between 2D MRCP and 3D MRCP image quality and the identification of the features of the cystic lesions.

	Image quality			Features of the cystic lesions		
		
	Read 1	Read 2	κ	Read 1	Read 2	κ
2D MRCP	3.51	3.49	0.508	3.26	3.31	0.696
3D MRCP	4.22	4.19	0.656	4.00	3.92	0.787
P-value	0.001	0.003		0.004	0.011	

Read 1 and 2, mean rank score. 2D, two-dimensional; 3D, three-dimensional; MRCP, magnetic resonance cholangiopancreatography.

**Table II:** Comparision between the visualization of the pancreatic main duct on 2D MRCP and 3D MRCP.

	Pancreatic duct visualization								
	
	Head			Neck			Tail		
			
	Read 1	Read 2	κ	Read 1	Read 2	κ	Read 1	Read 2	κ
2D MRCP	3.17	3.20	0.863	3.17	3.25	0.882	3.17	3.48	0.809
3D MRCP	3.37	3.26	0.848	3.17	3.19	0.713	3.11	3.33	0.806
P-value	0.210	0.660		0.363	0.782		0.117	0.314	

Read 1 and 2, mean rank score. 2D, two-dimensional; 3D, three-dimensional; MRCP, magnetic resonance cholangiopancreatography.

**Table III:** Comparision between the prediction of communication between the pancreatic main duct and cystic lesion made based on 2D MRCP and 3D MRCP.

	Az value		
	
	Read 1	Read 2	κ
2D MRCP	0.863	0.858	0.585
3D MRCP	0.923	0.892	0.779
P-value	0.616	0.671	

Read 1 and 2, mean rank score. 2D, two-dimensional; 3D, three-dimensional; MRCP, magnetic resonance cholangiopancreatography; Az, area under the receiver operating characteristic curve.
